# Specific knockout of kidney homogentisate 1,2-dioxygenase reveals that local metabolism of tyrosine and homogentisic acid is negligible in alkaptonuria

**DOI:** 10.1093/hmg/ddag036

**Published:** 2026-05-25

**Authors:** Dominic A Rutland, Brendan P Norman, Juliette H Hughes, Peter J M Wilson, Hazel Sutherland, Rebecca L W Brown, James A Gallagher, Lakshminarayan R Ranganath, George Bou-Gharios

**Affiliations:** Department of Musculoskeletal and Ageing Science, Institute of Life Course and Medical Sciences, University of Liverpool, 6 West Derby Street, Liverpool L7 8TX, United Kingdom; Department of Musculoskeletal and Ageing Science, Institute of Life Course and Medical Sciences, University of Liverpool, 6 West Derby Street, Liverpool L7 8TX, United Kingdom; Department of Musculoskeletal and Ageing Science, Institute of Life Course and Medical Sciences, University of Liverpool, 6 West Derby Street, Liverpool L7 8TX, United Kingdom; Department of Musculoskeletal and Ageing Science, Institute of Life Course and Medical Sciences, University of Liverpool, 6 West Derby Street, Liverpool L7 8TX, United Kingdom; Department of Musculoskeletal and Ageing Science, Institute of Life Course and Medical Sciences, University of Liverpool, 6 West Derby Street, Liverpool L7 8TX, United Kingdom; Department of Musculoskeletal and Ageing Science, Institute of Life Course and Medical Sciences, University of Liverpool, 6 West Derby Street, Liverpool L7 8TX, United Kingdom; Department of Musculoskeletal and Ageing Science, Institute of Life Course and Medical Sciences, University of Liverpool, 6 West Derby Street, Liverpool L7 8TX, United Kingdom; Department of Musculoskeletal and Ageing Science, Institute of Life Course and Medical Sciences, University of Liverpool, 6 West Derby Street, Liverpool L7 8TX, United Kingdom; Department of Musculoskeletal and Ageing Science, Institute of Life Course and Medical Sciences, University of Liverpool, 6 West Derby Street, Liverpool L7 8TX, United Kingdom

**Keywords:** tyrosine, kidney-specific knockout, metabolism, alkaptonuria

## Abstract

Extreme metabolic phenotypes present unique opportunities to understand the participation of different organs in specific metabolite pathways. One such condition is the inherited metabolic disorder alkaptonuria (AKU), caused by mutations in the gene encoding the homogentisate 1,2-dioxygenase (HGD) enzyme. HGD is expressed in liver and kidney. In AKU, lack of functional HGD results in incomplete breakdown of the amino acid tyrosine and accumulation of homogentisic acid (HGA), the indicative metabolite in AKU. Here, we aimed to delineate the role of the kidney in production and metabolism of HGA. We generated for the first time a mouse with specific deletion of *Hgd* in the kidney but not in the liver, using Cre recombinase driven by *Six2* (*Six2^GCiP^*), a transcription factor expressed early in kidney development. With intact liver HGD in this mouse, plasma HGA remained equivalent to wild-type concentrations. Minimal circulating HGA combined with no apparent renal HGD activity enabled us to unmask the portion of HGA produced locally within the kidney. Urine HGA (mean ± SEM) was > 100-fold lower in kidney-specific *Hgd* knockout mice (717 ± 129 μmol/L) compared to AKU mice (118 364 ± 8494 μmol/L) with complete liver and kidney knockout, but higher than wild-type controls (2.3 ± 0.1 μmol/L). Profiling of tyrosine pathway metabolic enzymes showed human and mouse kidney lack detectable tyrosine aminotransferase, a key enzyme involved in tyrosine-HGA metabolism. We demonstrate both tyrosine metabolism and HGA production are minimal in kidney compared to liver. The kidney is therefore not a viable target for HGA-lowering therapies aiming to restore HGD activity.

## Introduction

**Figure 1:**
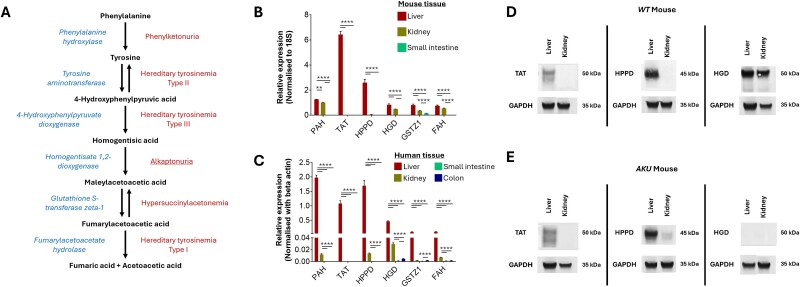
Expression of tyrosine metabolic enzymes in human and murine tissues. (A) Schematic diagram of phenylalanine/tyrosine metabolic pathway. Metabolic enzymes (left of pathway) are involved in the metabolism of phenylalanine/tyrosine to fumaric acid and acetoacetic acid via several intermediate metabolites including homogentisic acid (HGA). Deficiencies in functional expression of tyrosine metabolic enzymes results in a range of different metabolic diseases (right of pathway). (B) Relative expression of phenylalanine hydroxylase (PAH), tyrosine aminotransferase (TAT), 4-hydroxyphenylpyruvate dioxygenase (HPPD), homogentisate 1,2-dioxygenase (HGD), glutathione S-transferase zeta 1 (GSTZ1) and fumarylacetoacetate hydrolase (FAH) in wild-type (WT) mouse liver, kidney, small intestine and colon RNA extracts. Expression values normalised with 18S. (C) Relative expression of PAH, TAT, HPPD, HGD, GSTZ1 and FAH in human liver, kidney and small intestine. Expression values normalised with beta-actin. (D and E) western blotting analysis of TAT, HPPD and HGD protein expression in liver and kidney tissue of WT (D) and AKU (E) mice. GAPDH expression was used as a control. ^*^P < 0.05; ^**^P < 0.01; ^***^P < 0.001; ^****^P < 0.0001.

Tyrosine is a non-essential amino acid, that can be produced endogenously from metabolising phenylalanine, and is an important precursor for the synthesis of neurotransmitters, thyroid hormones, melanin, and body proteins [1, 2]. Tyrosine is predominantly metabolised in the liver by a series of enzymes (Fig. 1A). Deficiencies in the expression of these constituent metabolic enzymes result in a range of metabolic diseases, with differing incidences, severities, and phenotypes [3–5]. One of these diseases is alkaptonuria (AKU; OMIM #203500), an autosomal recessive disease caused by a deficiency of functional homogentisate 1,2-dioxygenase (HGD) [6, 7]. Normally, HGD metabolises homogentisic acid (HGA) to maleylacetoacetic acid. In AKU, non-functional or absent HGD results in high HGA concentrations in the circulation and urine [8]. Despite maximal urinary output of HGA, over many years HGA is deposited into the connective tissues, particularly the cartilage, as a dark brown pigment in a process called ochronosis [9, 10] leading to arthroplasty [8, 11, 12].

Organ specific gene deletion in mice by our laboratory has helped to reveal the contribution of the liver versus kidney to metabolism of HGA. Conditional knockout of liver *Hgd* but with intact kidney *Hgd* showed an increase in plasma HGA similar to levels observed in AKU mouse models (full *Hgd* knockout), indicating the importance of the liver in minimising circulating HGA concentrations. Despite plasma HGA concentrations in the typical range for AKU mice, urine HGA output in liver-specific *Hgd* knockout was only 12% of that observed in full *Hgd* knockout mice [13]; the reason for this remains unknown but could potentially reflect local metabolism of HGA in the kidney via intact renal *Hgd*.

In human AKU, lack of HGD activity in liver *and* kidney is believed to contribute to the generation of HGA, the fundamental molecule responsible for the pathogenesis of the condition. The exact contribution of the kidney versus the liver to HGA metabolism has never been characterised. In order to address this knowledge gap, we generated a new mouse model with normal liver *Hgd* expression but without kidney *Hgd.* This mouse provided the opportunity to ascertain the relative contribution of kidney HGD activity to circulating and urinary concentrations of HGA. We also went on to determine expression levels of the other tyrosine metabolic pathway enzymes in human and mouse kidney to develop our understanding of the role of this organ in tyrosine metabolism beyond HGA.

## Results

### Expression of tyrosine metabolic enzymes in kidney and other tissues

Comparison of the relative expression of genes encoding for tyrosine metabolic enzymes in human and mouse was carried out (Fig. 1B and C). In human total RNA extracts, expression of all enzymes, from phenylalanine hydroxylase (PAH) through to fumarylacetoacetate hydrolase (FAH), was higher in the liver than all other tissues analysed (*P* < 0.05; Fig. 1C). Comparison of tyrosine metabolic enzyme expression in mRNA extracted from C57BL/6 J WT mice (*n* = 3) showed similar trends to human RNA extracts (Fig. 1B). Notably, TAT expression was not detected in kidney, small intestine, or colon RNA extracts for human or mouse.

The difference in expression of PAH and FAH between liver and kidney was less marked in mouse (Fig. 1B) than in human extracts (Fig. 1C). Expression of HPPD in mouse kidney was minimal. In mouse small intestine, GSTZ1 (also known as maleylacetoacetate isomerase) was the only phe-tyr pathway for which we detected expression (Fig. 1B). We analysed tissue lysates by Western blot for TAT, HPPD and HGD and showed that protein content was consistent with level of mRNA expression (Fig. 1D-E).

### Kidney-specific Hgd knockout

To investigate the effect of kidney-specific deletion of *Hgd*, we first used a kidney-specific Cre recombinase expressed by *Pax8-CreER^T2^* (Supplementary Fig. 1A). We generated a double transgenic colony positive for *Pax8-CreER^T2^* and with a floxed *Hgd* gene (hereafter referred to as *Hgd*^fl/fl^  *Pax8-CreER^T2^* + ve; Supplementary Fig. 1B). Tamoxifen was administered to *Hgd*^fl/fl^  *Pax8-CreER^T2^* + ve mice (*n* = 4) for deletion of the *Hgd* gene. Control mice studied were *Hgd*-floxed *Pax8-CreER^T2^*-ve mice (*n* = 3), which were also administered tamoxifen.

**Figure 2:**
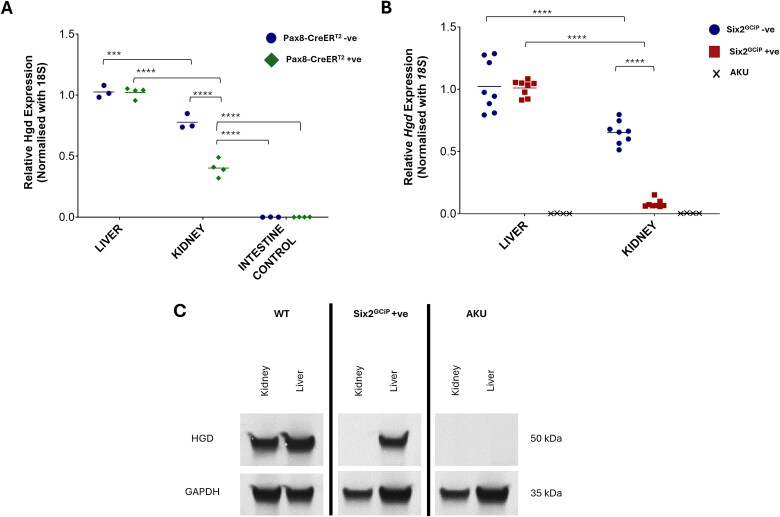
Analysis of *Hgd* mRNA and protein expression in kidney *Hgd* knockout mouse models. (A) Expression of *Hgd* mRNA in liver, kidney and small intestine in double transgenic *Hgd^fl/fl^ Pax8-CreER^T2^ +* ve (*n* = 4) and *Hgd^fl/fl^ Pax8-CreER^T2^-*ve controls (*n* = 4) mice following administration of tamoxifen. All expression values were normalised with *18S* expression. (B) Expression of *Hgd* mRNA in liver and kidney in double transgenic *Hgd*^fl/fl^  *Six2^GCiP(+/−)^* (*n* = 8), *Hgd*^fl/fl^  *Six2^GCiP(+/+)^* controls (*n* = 8) and full *Hgd* knockout AKU (*n* = 4) mice. All expression values were normalised with *18S* expression. (C) Expression of HGD protein in liver and kidney in double transgenic *Hgd*^fl/fl^  *Six2^GCiP(+/−)^*, *Hgd*^fl/fl^  *Six2^GCiP(+/+)^* controls and full Hgd knockout AKU mice. GAPDH protein expression was confirmed in all tissues. HGD and GAPDH expression was confirmed in multiple mice (*n* = 3), images are representative of the results. ^*^P < 0.05; ^**^P < 0.01; ^***^P < 0.001; ^****^P < 0.0001.

One week after the final tamoxifen dose, *Hgd^fl/fl^ Pax8-CreER^T2^ + ve* mice showed an approximate 50% reduction in kidney *Hgd* mRNA expression compared to controls (Fig. 2A). Liver *Hgd* expression showed no statistically significant difference between *Hgd*^fl/fl^  *Pax8-CreER^T2^ + ve* and control mice. *Hgd* expression was not detected in intestinal tissue extracts in *Pax8-CreER^T2^ + ve* or control mice (Fig. 2A).

PCR amplification of the *Hgd* gene in liver and kidney genomic DNA, using primers specific for both *Hgd* exons 5 and 7, was used to assess if Cre recombinase had excised the floxed exon 6 from *Hgd*. PCR results showed a truncated *Hgd* product limited to the kidney of Hgd^fl/fl^  *Pax8-CreER^T2^* + ve mice, with the intact *Hgd* gene product observed for controls (Supplementary Fig. 1C). Additionally, the genomic DNA from *Hgd^fl/fl^ Pax8-CreER^T2^ +* ve kidney showed some intact *Hgd* gene PCR product, indicating incomplete recombination of *Hgd* with tamoxifen treatment (Supplementary Fig. 1C). Increasing tamoxifen dose (to a maximum of 266 mg/kg body weight) or alternate route of administration (oral gavage) did not increase the deletion of kidney *Hgd* mRNA (data not shown).

**Figure 3:**
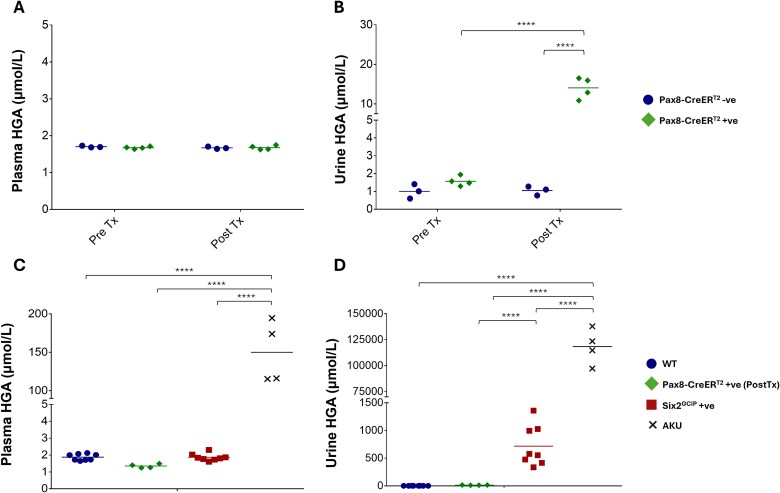
Metabolic analysis of HGA concentrations in serum and urine in *Hgd* knockout mouse models. (A & B) serum and urine HGA levels in *Hgd^fl/fl^ Pax8-CreER^T2^* mice respectively, before and after tamoxifen (Tx) injection. HGA concentrations were measured in *Hgd^fl/fl^ Pax8-CreER^T2^ +* ve (*n* = 4) and *Hgd^fl/fl^ Pax8-CreER^T2^-*ve control (*n* = 3). (C & D) HGA concentrations in serum and urine, respectively. HGA was measured in *Hgd*^fl/fl^  *Six2^GCiP(+/−)^* (*n* = 8), *Hgd*^fl/fl^  *Six2^GCiP(+/+)^* control (*n* = 8), and full *Hgd* knockout *AKU* (*n* = 4) and post Tx *Hgd^fl/fl^ Pax8-CreER^T2^ +* ve (*n* = 4). Normalisation of urine HGA to urine creatinine concentration had no observable impact on the directionality of the data. ^*^P < 0.05; ^**^P < 0.01; ^***^P < 0.001; ^****^P < 0.0001.

HGA was measured in plasma and urine samples collected before tamoxifen then one week after the final tamoxifen dose to determine the effect of kidney *Hgd* deletion. Mean (± SEM) plasma HGA concentration both pre- and post-tamoxifen in *Hgd^fl/fl^ Pax8-CreER^T2^ + ve* and control mice remained very low at 1.7 (±0.1) μmol/L, below the assay lower limit of quantification (LLOQ; 2.3 μmol/L) (Fig. 3A). Urine HGA in *Hgd^fl/fl^ Pax8-CreER^T2^ +* ve mice increased following tamoxifen to 14.1 (±1.3) μmol/L but remained below the assay LLOQ in controls both pre- and post-tamoxifen (Fig. 3B).

Since Cre driven by the *Pax8* promotor did not delete the *Hgd* gene sufficiently, we used a second Cre recombinase driven by *Six2* (*Six2^GCiP^*; Supplementary Fig. 1D), a transcription factor expressed earlier in nephron development than *Pax8* [14, 15]. *Six2^GCiP^* Cre expression did not require tamoxifen induction and resulted in deletion of the kidney *Hgd* gene at an earlier time point compared with *Pax8-CreER^T2^* (Supplementary Fig. 1E)*.* Double transgenic *Hgd*^fl/fl^  *Six2^GCiP(+/−)^* were generated carrying a homozygous floxed *Hgd* and heterozygous *Six2^GCiP^* Cre to delete kidney *Hgd* expression (Supplementary Fig. 1D). Control mice in this study were *Hgd*-floxed but wild-type for *Six2^GCiP^* Cre (*Hgd*^fl/fl^  *Six2^GCiP(+/+)^*).

Analysis of *Hgd* mRNA expression confirmed a reduction in kidney *Hgd* mRNA of > 90% in *Hgd*^fl/fl^  *Six2^GCiP(+/−)^* mice (*n* = 8) compared with *Hgd*^fl/fl^  *Six2^GCiP(+/+)^* controls (*n* = 8). The Hgd kidney expression in *Hgd*^fl/fl^  *Six2^GCiP(+/−)^* was therefore equivalent to our previously established AKU mouse with full *Hgd* knockout (p = 0.4849) (Fig. 2B).

PCR amplification of the *Hgd* gene from genomic DNA showed a truncated product in the kidney of *Hgd^fl/fl^ Six2^GCiP(+/−)^* mice due to removal of *Hgd* exon 6. The non-truncated, intact *Hgd* gene product only was observed for liver in *Hgd^fl/fl^ Six2^GCiP(+/−)^* and for liver and kidney in control *Hgd*^fl/fl^  *Six2^GCiP(+/+)^* mice. The residual non-truncated *Hgd* gene PCR product observed in *Hgd^fl/fl^ Six2^GCiP(+/−)^* mouse kidney was probably a result of incomplete recombination (Supplementary Fig. 1F).

Western blotting showed no detectable HGD protein in *Hgd*^fl/fl^  *Six2^GCiP(+/−)^* kidney, unlike control *Hgd*^fl/fl^  *Six2^GCiP(+/+)^* mice with normal kidney HGD. *Hgd*^fl/fl^  *Six2^GCiP(+/−)^* mice had equivalent liver HGD protein levels to control *Hgd*^fl/fl^  *Six2^GCiP(+/+)^* mice, showing specificity of our *Hgd* knockout to the kidney in *Hgd*^fl/fl^  *Six2^GCiP(+/−)^* (Fig. 2C). No HGD protein was detected in either liver or kidney tissue from full *Hgd* knockout AKU controls (Fig. 2C).

Mean (± SEM) plasma HGA was comparably low for *Hgd*^fl/fl^  *Six2^GCiP(+/−)^* (1.9 ± 0.1 μmol/L) and control *Hgd*^fl/fl^  *Six2^GCiP(+/+)^* mice (1.9 ± 0.1 μmol/L). For full *Hgd* knockout AKU mice, plasma HGA was in the typical range at (150 ± 20.3 μmol/L) (Fig. 3C) [13]. Despite the low plasma HGA in *Hgd*^fl/fl^  *Six2^GCiP(+/−)^*, mean urine HGA was 717 (± 129) μmol/L; 300-fold higher than in controls (2.3 ± 0.1 μmol/L) (*P* < 0.0001) but still approximately 165-fold lower than urine HGA in full *Hgd* knockout AKU mice (118 364 ± 8494 μmol/L) (Fig. 3D). Mean urine HGA was over 50-fold higher in *Hgd*^fl/fl^  *Six2^GCiP(+/−)^* compared with *Hgd^flf/fl^ Pax8-CreER^T2^ + ve* mice (*P* < 0.01).

In addition to HGA measurements, the upstream metabolites of HGA, from phenylalanine to HPPA, were quantified in urine and plasma (Supplementary Fig. 2). For plasma, no statistically significant difference was observed in these metabolites between *Hgd*^fl/fl^  *Six2^GCiP(+/−)^*, *Hgd*^fl/fl^  *Six2^GCiP(+/+)^* controls and full *Hgd* knockout AKU controls, except for plasma tyrosine, which was lower in *Hgd*^fl/fl^  *Six2^GCiP(+/−)^* mice with kidney specific *Hgd* deletion (82.1 ± 5.8 μmol/L) versus *Hgd*^fl/fl^  *Six2^GCiP(+/+)^* controls (102.9 ± 4.8 μmol/L) and full *Hgd* knockout AKU (106.2 ± 5.6 μmol/L) (p = 0.0372). HPPA and HPLA were the only urine metabolites with statistically significant differences between mouse genotypes; higher in full *Hgd* knockout AKU compared with both *Hgd*^fl/fl^  *Six2^GCiP(+/−)^* and *Hgd*^fl/fl^  *Six2^GCiP(+/+)^* (*P* < 0.0001).

**Figure 4:**
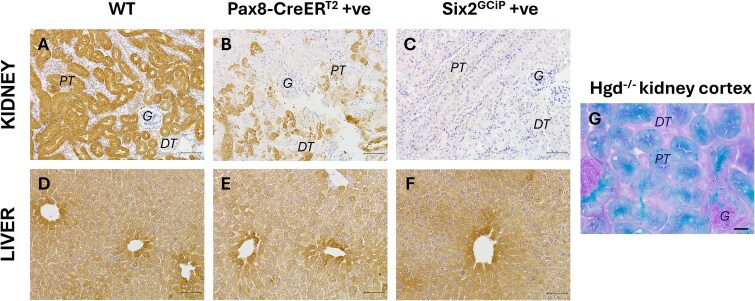
Immunohistochemical staining of tissue sections from kidney specific *Hgd* knockout mouse models. (A & D) DAB staining for HGD protein in paraffin wax kidney and liver sections for wild-type (WT) mice. HGD protein expression confirmed in proximal tubules (PT), and not in glomeruli (G) or distal tubules (DT). HGD protein expression confirmed throughout liver hepatocytes. (B & E) DAB staining for HGD protein in paraffin wax kidney and liver sections for *Hgd^fl/fl^ Pax8-CreER^T2^ +* ve mice. HGD protein expression shown to be decreased in PT cells, with no difference in liver sections compared with WT controls. (C & F) DAB staining for HGD protein in paraffin wax kidney and liver sections for *Hgd*^fl/fl^  *Six2^GCiP(+/−)^* mice. No HGD protein expression detected in any kidney structures, with no difference in liver sections compared with WT controls. (G) Staining for *LacZ* gene in knock-out first *Hgd^−/−^* frozen kidney sections, which shows positive blue staining for beta-galactosidase activity when provided with the substrate X-gal, combined with a periodic Schiff acid (PAS) stain for brush border membranes, confirming *LacZ* expression is localised to PT cells. *LacZ* expression confirms HGD expression is localised to PT cells in the kidney. All immunohistochemistry was carried out on multiple samples (*n* = 3), images are representative of the results. Scale bar (images A-F) = 50 μm. Scale bar (image G) = 25 μm.

Immunohistochemistry analysis of wild-type kidney sections showed strong, positive DAB staining for HGD protein in the proximal convoluted tubules (PCTs), with no HGD detected in the glomeruli, distal convoluted tubules of the cortex or in the renal medulla. These observations match those we reported previously in a *Hgd^−/−^* knockout first mouse model using a *LacZ* reporter gene [13] (Fig. 4G). Comparatively, the degree of staining in *Hgd^fl/fl^ Pax8-CreER^T2^ + ve* and *Hgd*^fl/fl^  *Six2^GCiP(+/−)^* kidney corresponded to the degree of *Hgd* gene knockout, with reduced HGD staining in *Hgd^fl/fl^ Pax8-CreER^T2^ + ve* and no positive staining detected for *Hgd*^fl/fl^  *Six2^GCiP(+/−)^* in kidney cortex (Fig. 4B and C). In contrast, staining of liver sections showed extensive and comparable HGD protein in all hepatocytes for all mice (Fig. 4D-F). Immunohistochemistry analysis of full *Hgd* knockout AKU kidney and liver, as well as rabbit IgG and no-primary-antibody controls on wild-type tissue sections showed no positive staining for HGD, with secondary antibody and DAB (Supplementary Fig. 3).

## Discussion

The present study demonstrates that the kidney plays a limited role in tyrosine metabolism and HGA production, thereby reinforcing the liver as the principal regulator of circulating HGA levels. These findings provide mechanistic support for liver-directed therapeutic strategies aimed at restoring HGD activity in AKU. Clinical observations are consistent with this conclusion since liver transplantation in patients with AKU markedly reduces plasma and urinary HGA concentrations, whereas kidney transplantation has no comparable effect [16, 17].

Both liver and kidney express HGD [16, 18] but the relative contributions of these organs to systemic HGA homeostasis have not previously been defined. Renal function is nonetheless critical in AKU due to the substantial excretion of HGA, which mitigates to some extent systemic accumulation. Impairment of renal clearance is associated with increased circulating HGA and accelerated ochronosis, contributing to disease morbidity [17]. While HGA itself does not appear intrinsically nephrotoxic, secondary complications, particularly renal calculi formation due to high urinary HGA concentrations, can result in renal impairment. In severe cases, acute kidney injury may precipitate systemic complications including oxidative haemolysis and methaemoglobinaemia [19, 20]. Within this context, defining the renal contribution to HGA production is essential. Our data indicate that this contribution is minimal.

Insights from related disorders of the phenylalanine/tyrosine pathway further support these findings. In hereditary tyrosinaemia type I (HT-1), toxic metabolite accumulation leads to severe hepatic injury, whereas renal involvement is comparatively less pronounced [21, 22]. This disparity is consistent with reduced metabolic flux through the pathway in the kidney, likely reflecting limited activity of key enzymes such as TAT in kidney, as we show here. Conversely, hereditary tyrosinaemia type II, characterised by inherited TAT deficiency, results in systemic tyrosinaemia but highlights a functional distinction between hepatic and renal metabolism: the absence of renal TAT permits conservation and reabsorption of tyrosine, while hepatic pathways govern its catabolism.

Extrapolating these observations, our findings suggest that the absence of renal TAT may represent an adaptive mechanism to preserve systemic tyrosine availability, particularly under conditions of fluctuating dietary intake. This concept is supported by evidence from chronic renal failure, where impaired tubular function reduces phenylalanine-to-tyrosine conversion, rendering tyrosine a conditionally essential amino acid [23]. The evolutionary basis and regulatory mechanisms underlying the absence of renal TAT remain unclear and warrant further investigation, including comparative analyses across species.

At the molecular level, our data demonstrate consistently higher expression of tyrosine pathway enzymes in human liver compared with the kidney. Notably, TAT was undetectable in both human and murine kidney at the mRNA and protein levels. In contrast, phenylalanine hydroxylase (PAH) was present in both human and mouse kidney, supporting previous reports that a substantial proportion of endogenous tyrosine production occurs in proximal convoluted tubules [24–26]. Together, these findings indicate that the kidney contributes to tyrosine synthesis but not its catabolism, instead favouring reabsorption and conservation. The absence of renal TAT appears to be conserved across species, including rodents and avian models [27, 28], further supporting a fundamental physiological role. We observed concordance between transcript and protein levels for the key enzymes we analysed for both gene (qPCR) and protein (western blot) expression, namely TAT, HPPD and HGD. However, for the other phenylalanine/tyrosine pathway enzymes, we cannot rule out that the pattern of gene expression may not directly relate to that of protein expression across the tissues we analysed.

Our study has important implications for kidney-specific gene targeting strategies. We observed that Six2-driven Cre recombinase achieved more efficient deletion of *Hgd* in proximal tubular cells than Pax8-driven Cre, likely reflecting earlier expression during nephrogenesis [14, 15]. In mice with kidney-specific *Hgd* deletion (Six2^GCiP^), plasma HGA concentrations remained comparable to wild-type controls, indicating that renal HGD is not required for systemic HGA regulation. This is consistent with previous findings that liver-specific *Hgd* deletion alone is sufficient to recapitulate the AKU phenotype [13]. Although urinary HGA was modestly elevated in kidney-specific knockout mice, levels were markedly lower than in global *Hgd*-deficient models, further underscoring the dominant role of hepatic metabolism.

The detection of low-level HGA in urine from kidney-specific *Hgd* knockout mice suggests limited local production despite the absence of TAT. One possible explanation is the activity of alternative aminotransferases, such as cytosolic and mitochondrial aspartate aminotransferases (GOT1/2), which may catalyse the conversion of tyrosine to HPPA. However, comparable levels of HPPA and HPLA between knockout and wild-type animals argue against a significant contribution from such alternative pathways.

In summary, our findings establish that the kidney contributes minimally to tyrosine catabolism and HGA production, with the liver serving as the central organ governing systemic HGA homeostasis. The absence of renal TAT supports a model in which the kidney prioritises tyrosine conservation rather than degradation. The functional significance of residual expression of downstream pathway enzymes, including HGD, in renal tissue remains unclear and may reflect additional, as yet uncharacterised roles. Further investigation into renal aminotransferases and the transport mechanisms of HGA may reveal novel kidney-based therapeutic targets for AKU and related metabolic disorders.

## Materials and methods

### Mouse husbandry and ethical approval

All mice were maintained and housed within the Biomedical Services Unit (BSU) at the University of Liverpool. Mice were housed in accordance with regulation from UK Home Office Guidelines, under specific pathogen-free conditions, *ad libitum* access to food and water and cage enrichment. Mice were maintained in an automatically controlled temperature and humidity environment, under a 12-hour light/dark cycle. All mice were culled by cervical dislocation. Work was carried out under project license PP8132802. The ARRIVE 2.0 reporting guidelines have been followed [29].

### Conditional kidney *Hgd* knockout mouse models

The conditional knockout of kidney *Hgd* was carried out first using *Pax8-CreER^T2^* transgenic Cre mice [30] (gift from Dr Laura Denby, University of Edinburgh, UK) mated with *Hgd^fl/fl^ (B6; B6N-Hgd^tm1(KOMP)Wtsi^*) to generate a double transgenic mouse (*Hgd^fl/fl^ Pax8-CreER^T2^ + ve*). Administration of tamoxifen was used to induce Cre recombinase activity via the modified oestrogen receptor (ER^T2^) [31–33]. At 8 weeks of age, *Hgd^fl/fl^ Pax8-CreER^T2^ + ve* (*n* = 4; 2 males & 2 females) and *Hgd*-floxed *Pax8-CreER^T2^*-ve controls (*Hgd*^*fl/*fl^; *n* = 3; 2 males & 1 female) mice were given tamoxifen via three intraperitoneal injections at 133 mg/kg body weight per injection, every two days. Plasma and urine samples were collected pre-tamofixen immediately before the first tamoxifen injection, and seven days after the final tamofixen dose. Liver, kidney and intestine were harvested for analysis after cull, seven days following the final tamoxifen dose.

A second kidney non-inducible conditional knockout was carried out using *Six2^GCiP^* knock-in Cre mice [34] (*B6.Six2^tm1(EGFP/cre)Phoh^*; gift from Peter Hohenstein, University of Leiden, Netherlands) which were mated with *Hgd^fl/fl^* mice to create a double transgenic model, *Hgd^fl/fl^Six2Cre^(+/−)^,* with *Hgd* conditionally deleted in nephron progenitor cells. Plasma and urine samples were collected from *Hgd^fl/fl^Six2Cre^(+/−)^* mice (*n* = 8; 5 males & 3 females) in addition to control mice without kidney *Hgd* deletion (*Hgd*-floxed but wild-type for *Six2^GCiP^* [*Hgd*^fl/fl^  *Six2^GCiP(+/+)^*]; *n* = 8; 3 males & 5 females) and full *Hgd^−/−^* knockout AKU mice [13] (*n* = 4; 3 males & 1 female). All mice were aged 8–12 weeks when culled. Liver and kidney were harvested post-mortem for mRNA, protein, and histological analysis.

The effect of kidney *Hgd* deletion was ascertained by plasma and urine concentration of tyrosine pathway metabolites (see biofluid metabolite quantification section), *Hgd* mRNA expression, and HGD protein presence (western blot and immunohistochemistry).

### Tyrosine pathway enzyme expression

Human total RNA extracts of liver, kidney, small intestine and colon were purchased (Takara Bio: 636531; ThermoFisher Scientific: QS0616, QS0626, QS0613) to determine the expression of tyrosine pathway enzymes. For mouse expression, liver, kidney and small intestine was isolated from C57BL/6 J wild-type mice (*n* = 3; 1 male & 2 females) and used for both mRNA and western blotting. Liver and kidney tissue from full *Hgd* knockout AKU mice (*n* = 4; 3 males & 1 female) were used for western blotting.

### RNA extraction and qPCR

Kidney, liver, and small intestine tissue samples from mice were homogenised using a liquid nitrogen-cooled pestle and mortar then divided for RNA and protein extraction (see western blotting below). RNA was extracted using QIAGEN RNeasy Mini Kit. Extracted murine RNAs, along with purchased human total RNAs (see Tyrosine pathway enzyme expression) were converted to cDNA using Applied Biosystems™ High-Capacity RNA-to-cDNA™ Kit (4387406) prior to qPCR.

Quantitative PCR (qPCR) was performed using synthesised cDNA diluted to a final working concentration of 5 ng/μl. Extension was carried out at 60°C for 30 seconds using primers shown in Supplementary Table 1.

### Western blotting

Tissues homogenates were lysed at 4°C in 0.2x radio-immunoprecipitation assay (RIPA) buffer. Lysates were sonicated, centrifuged at > 16 000x *g* for 20 minutes at 4°C, and the supernatant quantified for protein concentration using a bovine serum albumin (BSA) quantification assay. 30 μg of total tissue protein were loaded and ran on a 4–12% Bis-Tris polyacrylamide gel at 125 V for 90 minutes.

Proteins separated by SDS-PAGE were transferred from the polyacrylamide gel to a polyvinylidene fluoride (PVDF) membrane, blocked by 5% skimmed milk solution for 3 hours, and rabbit anti-HGD polyclonal antibody at 55 ng/ml (Proteintech; 16 465–1-AP) added to the membrane and incubated overnight at 4°C on a rocking platform. Goat-anti rabbit IgG conjugated alkaline phosphatase (AP) antibody (Promega; S3731) was then diluted (1 in 7500 in 1% w/v milk solution) and added to the membrane for 1 hour. After the final wash, the membrane was incubated with AP chromogenic substrate (ThermoFisher Scientific; WP20001) for 5 minutes. The membrane was imaged using the BioRad ChemiDoc XRS+ for the HGD protein (~50 kDa). PVDF membrane was also blotted for GAPDH as a control using goat anti-GAPDH antibody (Abcam; ab157156; 1 μg/mL) and donkey anti-goat Alexa Fluor® 488 secondary antibody (Abcam; ab150129; 400 ng/mL).

The same Westen blotting protocol as above was applied to detecting TAT and HPPD in WT and AKU mouse liver and kidney protein extracts. Rabbit-anti TAT recombinant antibody (Proteintech; 82 939–1-RR; 200 ng/μL) and rabbit anti-HPD polyclonal antibody (Proteintech; 17 004–1-AP; 90 ng/μL) were used for detecting TAT and HPPD respectively.

### Immunohistochemistry (IHC)

Kidney, liver, intestine/muscle tissue samples were fixed in 10% formalin for at least 24 hours, then stored in 70% ethanol. Paraffin wax tissue blocks were sectioned and slides were dewaxed and rehydrated, followed by incubation in H_2_O_2_ (Sigma Aldrich; H1009), and blocked with 5% v/v goat serum (Abcam; ab7481). Slides were incubated with rabbit anti-HGD primary antibody (Proteintech; 16 465–1-AP) diluted in 1% v/v goat serum at a concentration of 100 ng/ml at 4°C overnight. Slides were then incubated in goat ant-rabbit HRP conjugated secondary antibody (Vector Laboratories; MP-7451). The presence of HRP enzyme, and thus HGD protein, was detected using 3, 3′- diaminobenzidine (DAB) substrate (Vector Laboratories; SK-4100). Slides were then counterstained with Harris’ haematoxylin.

### Biofluid metabolite quantification

Blood samples were collected by tail vein puncture, and collected in lithium heparin microvettes (Sarstedt; CB 300 LH) and centrifuged at 1500x *g* for 10 minutes at 4°C. The plasma supernatant was acidified (protein crash) using 10% v/v 5.8 M perchloric acid and centrifuged at 2500x *g* for 10 minutes at 4°C. Urine samples were collected and acidified with 5% v/v 1 M sulfuric acid.

Phenylalanine/tyrosine pathway metabolites phenylalanine, tyrosine, 3(4-hydroxyphenyl)pyruvic acid (HPPA), 3-(4-hydroxyphenyl)lactic acid (HPLA) and HGA were quantified in urine and plasma using published liquid chromatography tandem mass spectrometry (LC–MS/MS) assays [35–37]. Creatinine was additionally measured in urine samples using LC–MS/MS. Metabolite measurements were carried out on a 1290 Infinity II liquid chromatography (LC) system coupled to a triple quadrupole mass spectrometer. Reversed-phase LC was performed on an Atlantis dC18 column (100 mm x 3 μm, 3 μm; Waters, UK). Mobile phases were (A) water and (B) methanol, both containing 0.1% v/v formic acid and flow rate 0.4 mL/min across for assays.

### Statistical analysis

The *in vivo* work was unblinded, as researchers were aware of the experimental groups. Only mass spectrometry analysis was blinded. No mice, samples, or data points were excluded from the analysis. Two-way ANOVA and one-way ANOVA analyses were carried out on mRNA and LC–MS/MS data respectively using PRISM GraphPad (v.6.05). All data met the assumptions of the above statistical tests.

## Supplementary Material

Supplementary_Material_ddag036
