# Supplementary material for: Specific knockout of kidney homogentisate 1,2-dioxygenase reveals that local metabolism of tyrosine and homogentisic acid is negligible in alkaptonuria
Source: Hum Mol Genet. 2026 May 25;35(8):ddag036. doi: 10.1093/hmg/ddag036 (PMC13200543; doi:10.1093/hmg/ddag036)
Supplement: Supplementary_Material_ddag036 [file supplementary_material_ddag036.docx]

**Supplementary Table 1** – Sequences for primers used in qPCR analysis of tyrosine metabolic enzyme expression including housekeeping gene sequences and primer binding efficiencies.

| **Target Gene** | **Forward Primer** | **Reverse Primer** | **Primer Efficiency** |
| --- | --- | --- | --- |
| Human PAH | 5’- ATGTCCACTGCGGTCCTG | 5’- GCCAATGCACCAACTTCTTC | 108% |
| Human TAT | 5’- GGGACCCTACTGTGTTTGGA | 5’- TAGCTTCTAGGGGTGCCTCA | 103% |
| Human HPPD | 5’- CAACCCCTGGAACAAAGAGA | 5’- CCTTCCCAAACTTGTCTTGC | 107% |
| Human HGD | 5’- ACAAGCCCTTTGAATCCAT | 5’- TATGCAGGCCACTCACAAAG | 103% |
| Human GSTZ1 | 5’- GGGGAAGCCCATCCTCTAT | 5’- TGGCACCTGCTTCATAGGAT | 102% |
| Human FAH | 5’- CAGCAGCTCACTCACCACTC | 5’- CTGACCATTCCCCAGGTCTA | 108% |
| Human Beta-actin | 5’- GGACCTGACTGACTACCTC | 5’- GCCATCTCTTGCTCGAAG | 98% |
| Mouse Pah | 5’- AAGACAGCCTGCCAGGAGTA | 5’- CCCTTTGAGTGTAGGGGTCA | 102% |
| Mouse Tat | 5’- TTAAGTCCAATGCGGACCTC | 5’- GCTCTGTGAATTCCACGTCA | 98% |
| Mouse Hppd | 5’- ATCGCTCTCAAGACGGAAGA | 5’- TGAGATTCTCCCGAAGCAGT | 97% |
| Mouse Hgd | 5’- GACCCATCGGAGCAAATGGC | 5’- AGTGTAACCACCTGGCACTC | 96% |
| Mouse Gstz1 | 5’- CCGGGAAGCCTATCCTCTAC | 5’- ACTTGCTTCATGGGGTTCAG | 109% |
| Mouse Fah | 5’- GCCAGCCCTACACATTTGAT | 5’- GGTCTCAGGTTGCATCCATT | 101% |
| Mouse 18S | 5’- GAAAATAGCCTTCGCCATCA | 5’- AGTTCTCCAGCCCTCTTGGT | 98% |

**
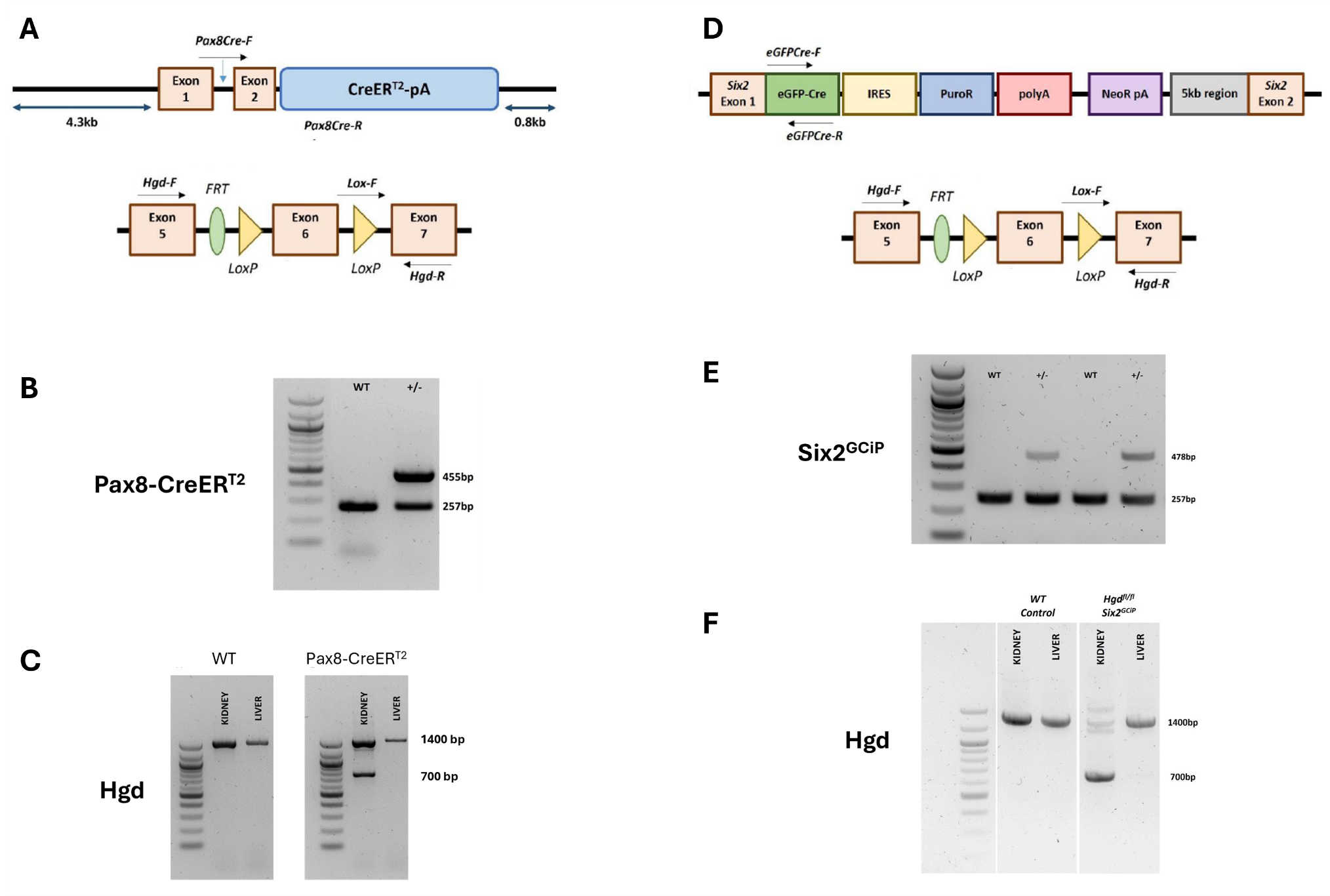
Supplementary Figure 1 Genotyping of kidney-specific *Hgd* knockout mouse models and post Cre recombination of kidney *Hgd* gene.** (A) Schematic of *Pax8-CreER^T2^* and *Hgd tm1c* transgenes including locations of primer sequences for genotyping to confirm presence of Cre recombinase and for kidney DNA after Cre recombination. (B) Genotyping for expression of *Pax8-CreER^T2^* Cre recombinase. Positive *Pax8-CreER^T2^*  mice produce a 455bp band, produced by *Pax8Cre-F* (5’-GTATGTCACCTAGGGGCTAGAAGGGAGCTG) and *Pax8Cre-R* (5’-CTCATCACTCGTTGCATCGACCGGTAATGC) primers. *Lox-F* (5’-GAGATGGCGCAACGCAATTAATG) and *Hgd-R* (5’-CTTGGCTTTGGACTTGAACCCTAGC) primers used to for *Hgd tm1c* genotyping used a positive control to confirm PCR results in *Pax8-CreER^T2^* negative mice. (C) Genotyping of *Hgd* in kidney genomic DNA using *Hgd-F* (5’-GCCTTTATCCCTACAAGTCTCCCCG) and *Hgd-R* primers. *WT* kidney and liver DNA only contain non-recombined *Hgd tm1c* gene, producing a 1400bp band. *Hgd^fl/fl^* *Pax8-CreER^T2^+*ve liver DNA shows only a 1400bp band like that of *WT* mice and confirms Cre recombinase activity is not present in the liver. Kidney DNA from *Hgd^fl/fl^* *Pax8-CreER^T2^+*ve mice shows evidence of a truncated PCR product (700bp) due to Cre recombination of the *Hgd tm1c* transgene, although the previously described 1400bp band showing *Hgd* recombination is not complete. (D) Schematic of *Six2^GCiP^* and *Hgd tm1c* transgenes including locations of primer sequences for genotyping to confirm presence of Cre recombinase and for kidney DNA after Cre recombination. (E) Genotyping for expression of *Six2^GCiP^* Cre recombinase. Positive *Six2^GCiP^* mice produce a 455bp band, produced by *eGFPCre-F* (5’- ACACCCTGGTGAACCGCATC) and *eGFPCre-R* (5’- CTCATCACTCGTTGCATCGACCGGTAATGC) primers. *Lox-F* and *Hgd-R* primers used to for *Hgd tm1c* genotyping used a positive control to confirm PCR results in *Six2^GCiP^* negative mice. (F) Genotyping of *Hgd* in kidney genomic DNA using *Hgd-F* and *Hgd-R* primers. *WT* kidney and liver DNA only contain non-recombined *Hgd tm1c* gene, producing a 1400bp band. *Six2^GCiP^* *+ve* murine liver DNA shows only a 1400bp band like that of *WT* mice and confirms Cre recombinase activity is not present in the liver. Kidney DNA from *Hgd*^fl/fl^ *Six2^GCiP(+/-)^* mice shows evidence of a truncated PCR product (700bp) with strong Cre recombination of the *Hgd tm1c* transgene. Some residual 1400bp band is present in the kidney DNA but likely due to non-progenitor derived kidney cells, such as collecting ducts and renal calyces/pelvis.

**
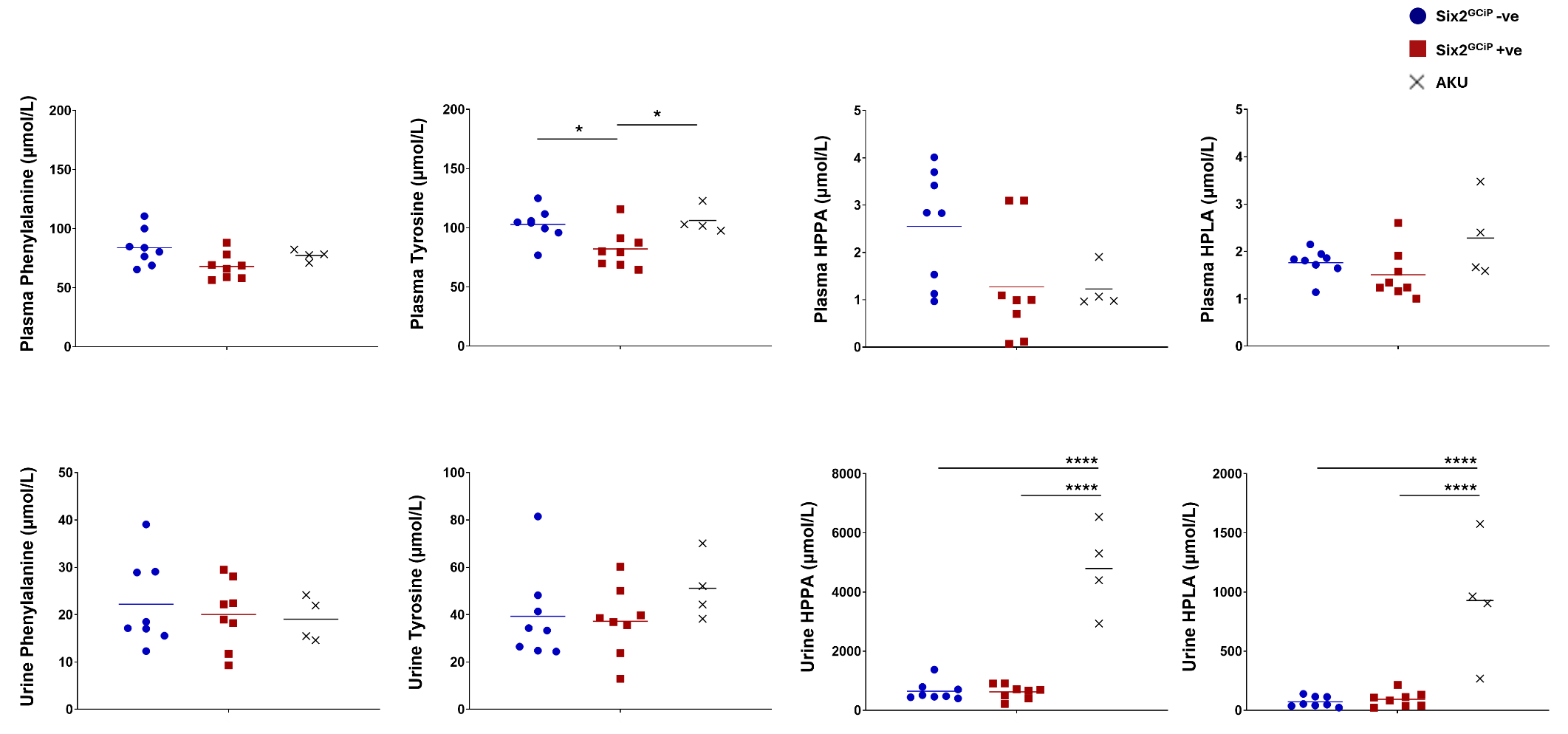
**

**Supplementary Figure 2 Metabolic analysis of phenylalanine/tyrosine metabolites in *Hgd* knockout mouse models.** Serum and urine levels of phenylalanine, tyrosine, HPPA and HPLA in *Hgd*^fl/fl^ *Six2^GCiP(+/-)^* (n=8), *Hgd*^fl/fl^ *Six2^GCiP(+/+)^* (n=8) and full *Hgd* knockout AKU (n=4) mice.

**
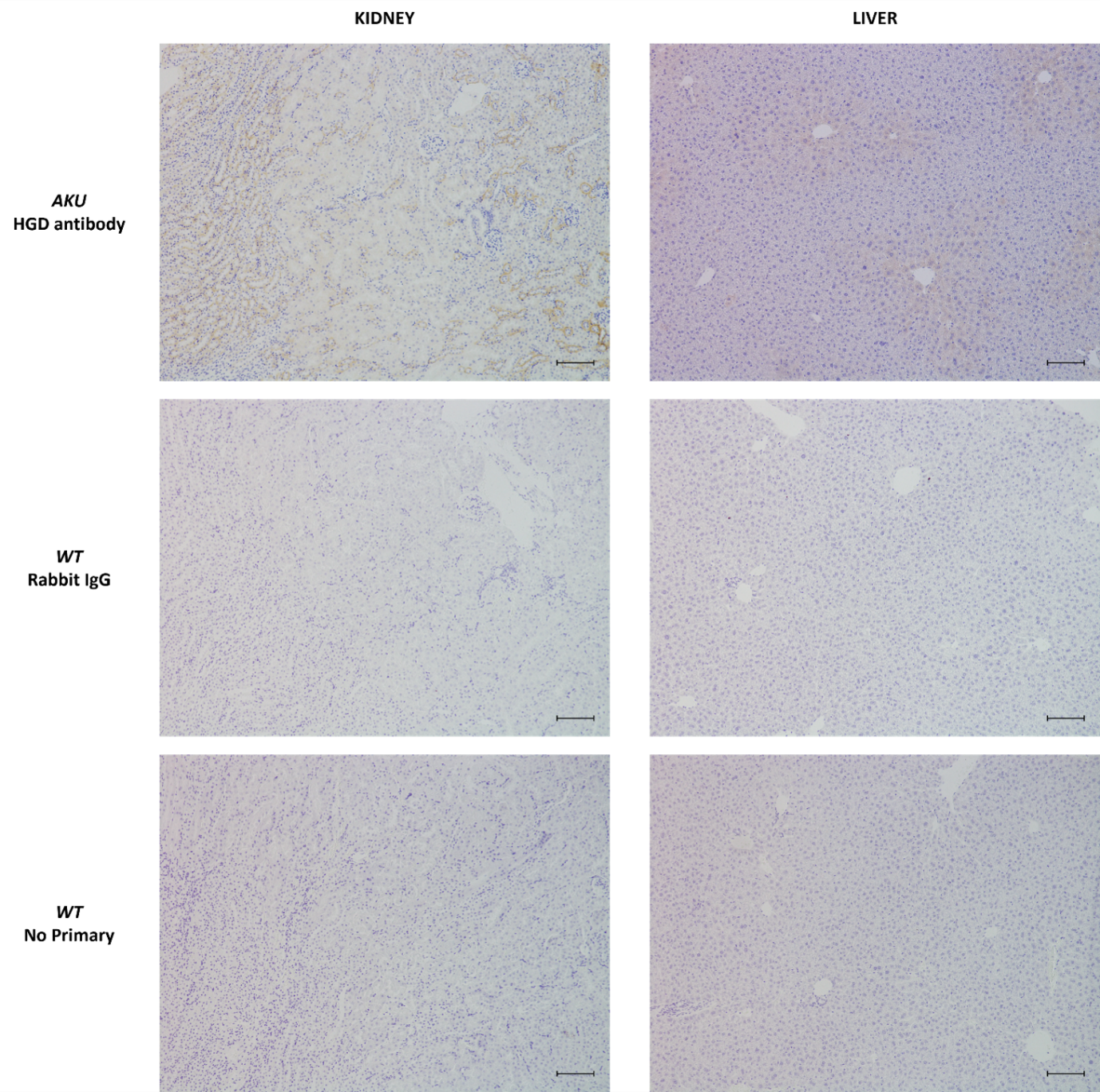
**

**Supplementary Figure 3 Immunohistochemical controls for kidney-specific *Hgd* knockout mice.** Immunohistochemistry of *AKU* kidney and liver sections showed no evidence of DAB staining due to complete knockout of *Hgd* gene. *WT* kidney and livers sections incubated with rabbit IgG and no primary antibody respectively showed no DAB staining confirming only positive signals were due to HGD antibody binding to HGD protein. Scale bar (all images) = 100 μm.
